# Tripterygium Ingredients for Pathogenicity Cells in Rheumatoid Arthritis

**DOI:** 10.3389/fphar.2020.583171

**Published:** 2020-10-02

**Authors:** Yujun Tang, Qiuping Liu, Yuxiang Feng, Yi Zhang, Zhenghao Xu, Chengping Wen, Yun Zhang

**Affiliations:** College of Basic Medical Science, Zhejiang Chinese Medical University, Hangzhou, China

**Keywords:** Tripterygium wilfordii Hook f., rheumatoid arthritis, immune cell, mechanism, review

## Abstract

Rheumatoid arthritis (RA) is an autoimmune disease mainly characterized by chronic polyarthritis. Many types of cells play pivotal roles in the pathogenicity of RA, such as T cells, B cells, macrophages, dendritic cells (DCs), osteoclasts (OCs), and fibroblast-like synoviocytes (FLS). Tripterygium wilfordii Hook f. (TwHf) and its ingredients are able to control disease activity by regulating the functions of cells mentioned above, and the clinical studies have highlighted the importance of TwHf ingredients in RA treatment. They have been demonstrated to improve the RA symptoms of animal models and patients. In this review, we discussed the effect of TwHf ingredients on pathogenicity cells, including disease/cell phenotypes and molecular mechanisms. Here, we constructed a cell-cell interaction network to visualize the effect of TwHf ingredients. We found that TwHf ingredients could inhibit the differentiation and proliferation of the pathogenicity cells. Besides, the components could decrease the levels of pathogenicity cytokines [i.e., interleukin-6 (IL-6), interleukin-1β (IL-1β), and tumor necrosis factor-α (TNF-α)]. Many signaling pathways are involved in the underlying mechanisms, such as PI3K, NF-κB, and MAPK signaling pathways.

## Introduction

Rheumatoid arthritis (RA) is an autoimmune disease mainly characterized by chronic persistent synovitis, which causes the destruction of articular cartilage and bone, eventually leading to joint deformity and finally loss of function. The incidence of RA in mainland China is about 0.42%, and the disability rate of this disease course over 15 years is 61.3%. The clinical studies have shown that the effectiveness of the anchoring drug methotrexate is only 15%–25% (Lopez‐Olivo et al., 2014; Chinese Rheumatology Association, 2018). While the addition of Tripterygium wilfordii Hook. f. (TwHf) or Tripterygium hypoglaucum (Levl.) Hutch is able to control RA disease activity more effectively by regulating immune cell functions (Jun et al., 2016; Xiao-yue et al., 2019). In the separable components of TwHf and Levl. Hutch, there are many similar but different active component, such as Wilforlide, Celastrol (Cel), and Triptolide (TP) (Xianguang and Li, 2006; Chao, 2015; Chen-qiong et al., 2015). These active components can regulate the pathogenicity immune cells and connective tissue cells of RA. For example, they can reduce the secretion of inflammatory cytokines (such as TNF-α, IL-1β, and IL-6) of macrophages, the proliferation, and differentiation of pathogenic T cells, and the bone destruction which mediated by the fibroblast-like synoviocytes (FLS) and the osteoclasts (Peng et al., 2014; Wang et al., 2014; Te et al., 2019).

Tripterygium genus alleviates disease activity by regulating RA-related cells by multiple targeting approaches. Up to date, there is no literature review for TwHf or its active ingredients on RA-related cell dysfunction. Meanwhile, there are signal transduction interactions between various immune cells and connective tissue cells in the process of RA, which jointly promote the occurrence and development of RA. Therefore, we conducted a literature review (search strategy is available in **Supplementary Materials 1**) to summarize the effects of Tripterygium genus active ingredients on RA-related cells. Furthermore, we constructed signal pathways and cell-cell interaction networks to summarize their molecular mechanisms and to speculate the potential target cells and proteins.

## The Functions of Tripterygium Ingredients on T Cells and the Molecule Mechanisms

T cells play a crucial role in various adaptive immune responses. During RA, T cells received antigens will be activated and proliferate (Smolen et al., 2018). Ho et al. (2013) found that PG27, one of the ingredients of TwHf, inhibited the T cell activation *via* targeting NF-κB and AP-1 pathways. PG27 can inhibit IKKα/IκBα/NF-κB and mitogen-activated protein kinase (MAPK)-AP-1 signaling pathways, while IKKβ activity was less sensitive for the inhibition of PG27. By contrast, the purified component of TwHf, PG490 (triptolide), similarly suppressed the above pathways. Similar results were demonstrated in RA animal models and patients but lacking molecule mechanisms. Triptolide reduced the numbers of CD4+ cells in the periphery and increased the numbers of CD8+ cells in Peyer’s patch (Zhou et al., 2006). When triptolide was used to treat T cell isolated from peripheral blood of RA patients, the percentage of CD4+ and CD8+T cells secreting IFN-γ, IL-2, and IL-4 was decreased, and the percentage of CD4+ and CD8+T cells expressing CD69 and CD25 was also reduced (Ming et al., 2014). Besides, Tripterygium active compounds have been demonstrated *in vivo* and *in vitro* to reduce T cell number by promoting T cell apoptosis as well as suppressing T cell proliferation and cytokine secretion, while the mechanism is unknown (Tao et al., 1991; Cascão et al., 2015b; Wang et al., 2018).

CD4+ T cells can activate and polarize into various T helper cell subsets, including T helper 1 (Th1), T helper 2 (Th2), regulatory T (Treg), T helper 9 (Th9), T follicular helper cells (Tfh), T helper 17 (Th17), or T helper 22 (Th22) cells. Th17 cell numbers were increased in the peripheral blood, inflamed synovial tissue, and synovial fluid of RA patients (Leipe et al., 2010; van Hamburg et al., 2011; Penatti et al., 2017). Th17 cells promote the development of RA through the secretion of various inflammatory cytokines and chemokines. TGF-β/SMADs/RORγt and IL-6/STAT3 pathways are involved in mediating Th17 cell differentiation and mediating the expression of IL-17A, IL-17F, and IL-21 (Ivanov et al., 2006; Nishihara et al., 2007; Yang et al., 2008). The Cel, one of the Tripterygium ingredients, has been proved to have anti-arthritic activity by inhibiting IL-6/STAT3 signal and finally reduce the secretion of Th17-related pro-inflammatory cytokines (Venkatesha et al., 2011). Moreover, Cel inhibits the activation of NF- κB, and caspase-1 in macrophages, resulting in the reduced release of IL-1β and TNF-α, and finally decreased the infiltration and proliferation of joint Th17 cells (Cascão et al., 2012) because IL-1β is able to promote the polarization of Th17 cells through inducing the expression of the transcription factors IFR4 and RORγ (Vallières et al., 2019). In addition, TP inhibits the expression of COX2 and the secretion of PGE2 in the co-culture models of RA synovial fibroblasts (RASFs) and RA CD4^+^ T cells, blocking the differentiation of Th17 cells *in vitro* (Peng et al., 2014).

Similar to Th17, Tfh cells also promote RA progression by secreting IL-21 (Vinuesa et al., 2016). However, there is less research on the effects of TwHf on Tfh. In patients with RA treated with TwHf, the number of tenderness joints, the number of swollen joints, and the evaluation score of overall RA in the experimental group were lower than those in the control group. Consistently, the levels of Tfh cells and IL-21 were lower than those in the control group, and the levels of Tfh cells and IL-21 were positively correlated with DAS28 score (Sun et al., 2016).

Treg cells act as protective cells during RA. Enhancing the function or improving the number of Treg cells has been proved to alleviate the RA activity in varying degrees (Cooles et al., 2013). So far, research focused on the effects of TwHf on Treg cells in RA were limited. In the co-culture system of bone marrow macrophages and Tregs, TP up-regulated IL-10 and TGF-β1 produced by Treg cells, resulting in the inhibition of osteoclast differentiation and bone resorption (Xu et al., 2016).

**Figure 1:**
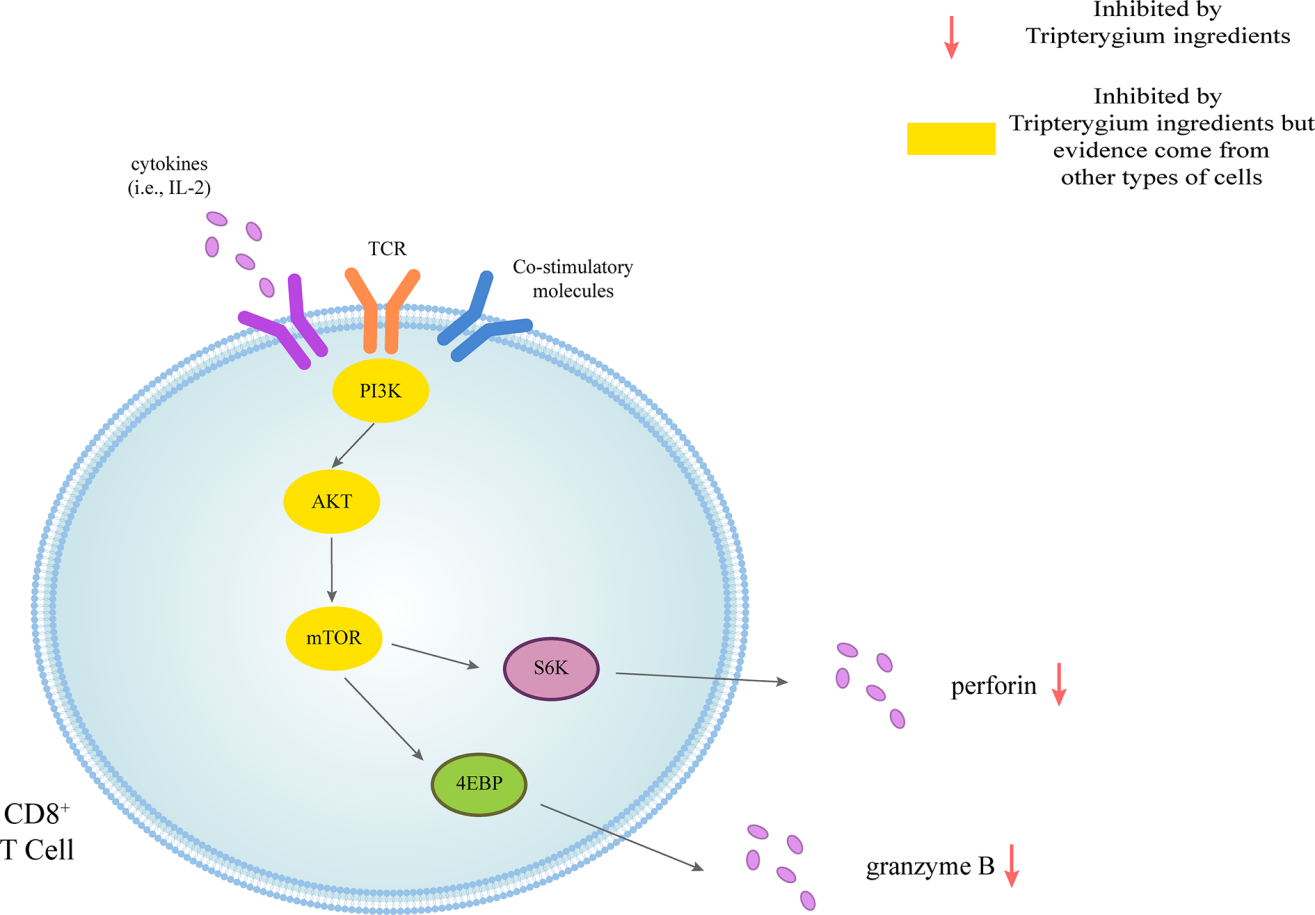
Tripterygium ingredients act on the CD8^+^ T cell. CD8^+^ T cells could be activated through three signals. The first signal of T cell activation comes from the specific binding of its receptor TCR to the antigen. The second signal of T cell activation comes from costimulatory molecules. Cytokines promote the full activation of T cells. Perforin and granzyme B are secreted to exert cellular immunity *via* the PI3K signaling pathway. In other cell types, tripterygium ingredients act on the PI3K signaling pathway to prohibit the activation of CD8^+^ T cells. Note: AKT (also known as PKB), Protein kinase B; PI3K, Phosphoinositide 3-kinase; mTOR, Mechanistic target of rapamycin kinase; S6K, Ribosomal protein S6 kinase; TCR, T-cell receptor; 4EBP, 4E-binding protein.

**Figure 2:**
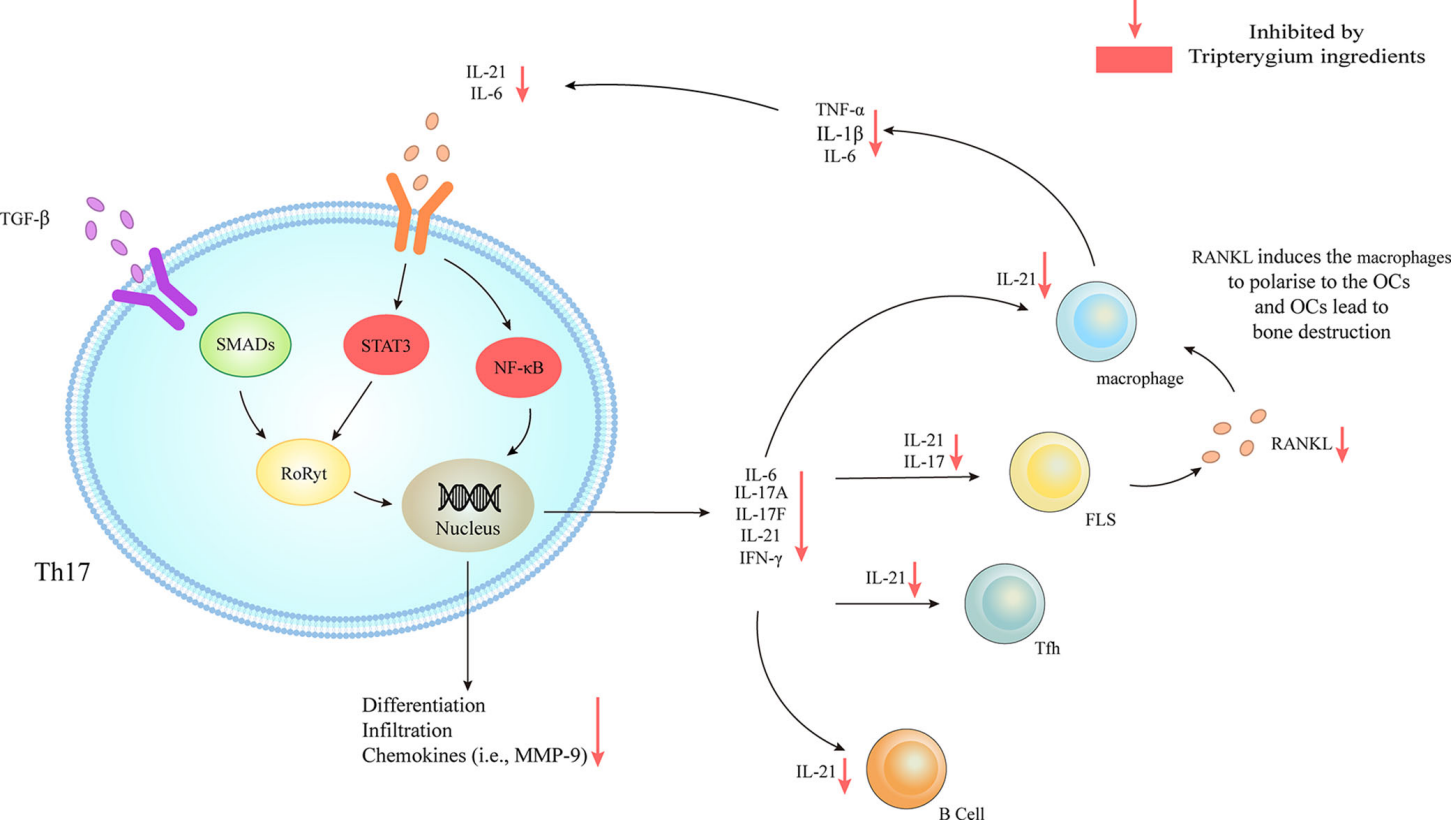
Tripterygium ingredients act on Th17. TGF-β/SMADs/RORγt, IL-6/STAT3/RORγt, and IL-6/NF-κβ signaling pathways are responsible for the differentiation and the secretion of chemokines and cytokines of Th17. The downstream effector cells include B cells, Tfh, FLS, and macrophages. The activation of FLS releases RANKL to promote the secretion of chemokines (i.e., IL-6, IL-1β, and TNF-α) of macrophages, which could target Th17 and further active Th17. Tripterygium ingredients inhibit STAT3 and NF-κβ, resulting in the reduction of many cytokines (i.e., IL-6 and IL-21) to negatively regulates Th17 and downstream effector cells. Note: FLS, Fibroblast-like synoviocytes; IFN-γ, Interferon-gamma; IL, Interleukin; NF-κβ, Nuclear factor-kappa B; RANKL, Receptor activator of nuclear factor-κB ligand; RORγt, Retinoic acid-related orphan receptor gamma t; SMAD, Suppressor of mothers against decapentaplegic; STAT, Signal transducer and activator of transcription; TGF-β, Transforming growth factor-beta; TNF-α, Tumor necrosis factor-alpha.

The role of tripterygium ingredients for T cells and the molecule mechanisms were summarized in **Table 1**. The molecule mechanisms of CD8^+^ cell and Th17 are available in **Figures 1**, **2** (Th1, Th2, Treg, and Tfh are not available because insufficient study describes their molecule mechanism).

**Table 1:** The effects of Tripterygium ingredients on T cells.

Subtype	Component	Models	Molecular mechanism	Effects	Animal disease phenotype	Ref.
CD4^+^T cell	TP	CIA rats	NA	Reduce the number of CD4^+^ T cells in periphery blood	Ameliorate	(Zhou et al., 2006)
CD8^+^ T cell	TP	CIA rats	NA	Increase the number of CD8^+^ T cells in Peyer’s patch	Ameliorate	(Zhou et al., 2006)
CD4^+^T cell and CD8^+^T cell	TP	Peripheral blood T cells in RA patients	NA	Reduce the percentage of CD4^+^ and CD8^+^ T cells. Reduce the levels of IFN-γ, IL-2, IL-4. Decrease the expression of CD69 and CD25	NA	(Ming et al., 2014)
Th17	Cel	AIA Lewis rats	Prohibit the phosphorylation of STAT3 and ERK	Inhibit the differentiation of Th17; decrease the levels of cytokines (IL-17, IL-6, and IFN-γ) and antibodies (anti-Bhsp65 and anti-CCP)	Ameliorate	(Venkatesha et al., 2011)
Th17	TP	Synovial fibroblasts from RA patients and Th17 cells co-cultured model	regulating cyclooxygenase-2/prostaglandin E2 axis	Inhibit Th17 differentiation	NA	(Peng et al., 2014)
Th17	Cel	E. coli stimulated THP-1 macrophage-like cell line and AIA Wistar rats	Inhibiting activation of NF-κB and caspase-1	Inhibit the release of IL-1β and TNF from macrophages, reducing joint the infiltration and proliferation of TH17 cells	Ameliorate	(Cascão et al., 2012)
Treg	TP	Co-cultures system of Tregs and BMMs	NA	Up-regulate IL-10 and TGF-β1, secreted by Treg. Inhibit the osteoclast differentiation and bone resorption caused by osteoclast	NA	(Xu et al., 2016)
TfH	Tripterygium glycosides tablets	Peripheral blood in patients with RA	NA	Decrease the numbers of TfH and the levels of IL-21	Ameliorate	(Sun et al., 2016)
T cell	PG27	Human peripheral blood T cells	Inhibiting activation of IKKα and AP-1	Inhibit the activation of T cell	NA	(Ho et al., 2013)
T cell	TP	Human peripheral blood T cells	Inhibiting activation of IKKα, AP-1, and IKKβ	Inhibit the activation of T cell	NA	(Ho et al., 2013)
T cell	Cel	AIA rats	NA	Promote T cell apoptosis	Ameliorate	(Cascão et al., 2015b)
T cell	TP	TNF transgenic mice	NA	Promote T cell apoptosis	Ameliorate	(Wang et al., 2018)
T cell	Triptolide ethanol extraction	Human peripheral blood	NA	Inhibit antigen and mitogen stimulated T cell proliferation and the secretion of IL-2	NA	(Tao et al., 1991)

Cel, Celastrol; TP, Triptolide; STAT3, Signal Transduction and Transcription Activator Protein 3; SOCS, Cytokine Signal Negative Regulator; IL, Interleukin; IKK, IκB Kinase; AP-1, Activin 1; TfH, Follicular helper T cells; OC, Osteoclasts; TNF, Tumor necrosis factor.

## The Effects of Tripterygium Ingredients on B Cells

**Figure 3:**
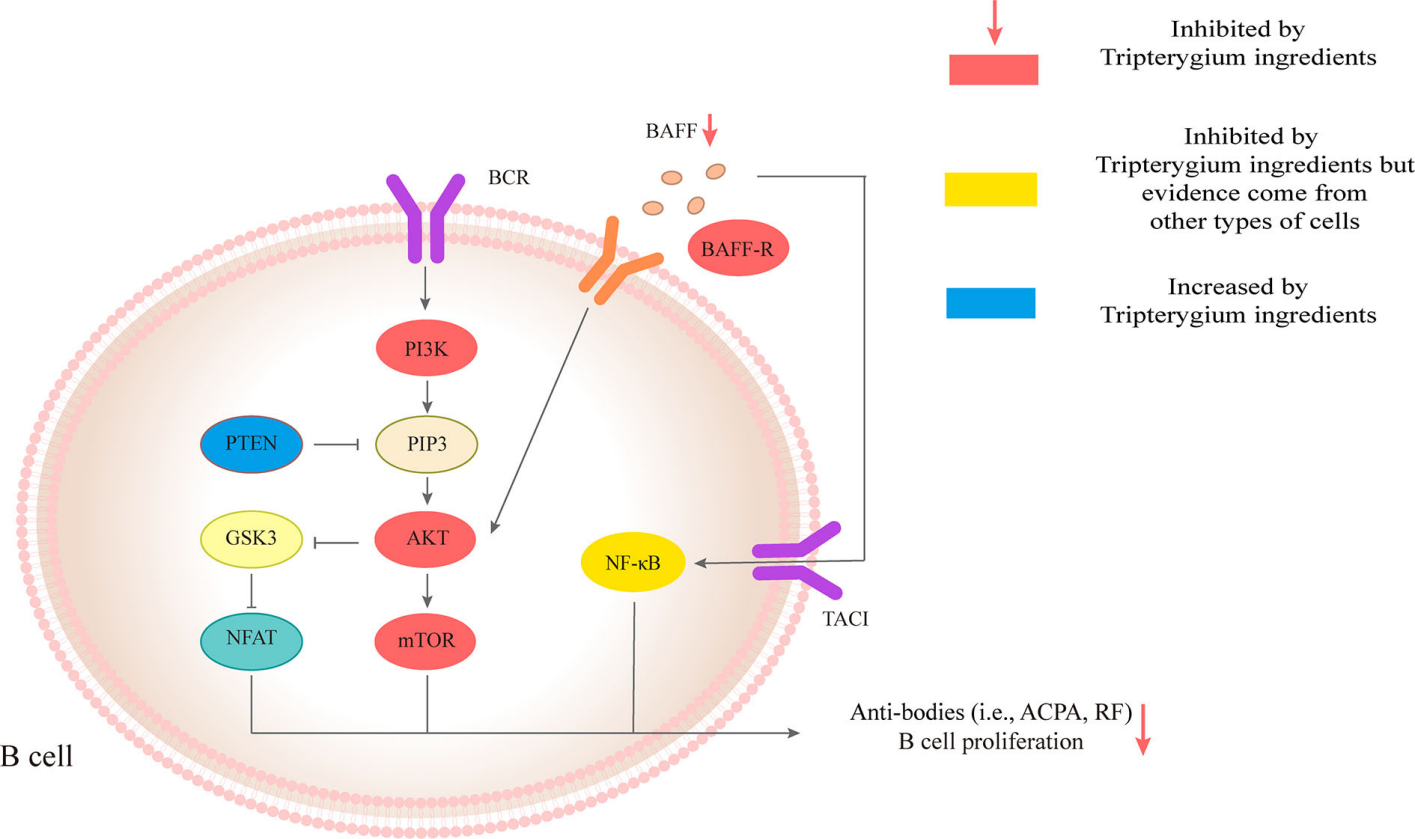
Tripterygium ingredients act on the B cell. The anti-bodies production and proliferation of B cells are associated with BAFF/AKT/mTOR, PI3K/AKT/mTOR, and TACI/NF-κβ signaling pathways. PTEN negatively regulates PI3K/AKT/mTOR signaling pathways through dephosphorylating the lipid signaling intermediate PIP3. Tripterygium ingredients could increase the levels of PTEN and decrease the levels of molecules (red nodes) in the three pathways mentioned above to inhibit the anti-bodies production and proliferation of B cells. Note: AKT (also known as PKB), Protein kinase B; BCR, B cell receptor; BAFF, B cell-activating factor; BAFF-R, B cell-activating factor receptor; GSK3. Glycogen synthase kinase-3; mTOR, Mechanistic target of rapamycin kinase; NF-κβ, Nuclear factor-kappa B; NFAT, Nuclear factor of activated T-cells; PI3K, Phosphoinositide 3-kinase; PIP3, phosphatidylinositol-3,4,5-trisphosphate; PTEN, Phosphatase and tensin homolog.

B cells are also critical in the development of RA. B cells can be used as antigen-presenting cells (APC) to provide synergistic stimulation and then activate T cells. In addition, B cells are able to secrete autoantibodies, such as rheumatoid factor (RF) and anti-citrullinated protein antibody (ACPA) (Scherer et al., 2018; Smolen et al., 2018). ACPAs, RF, or their immune complexes interact with immune cells such as macrophages, neutrophils, and osteoclasts to promote joint inflammation of RA. The differentiation and activation of B cells could be mediated by BAFF/BAFF-R-ATK-mTOR, and TACI-NF-κB (Niiro and Clark, 2002; Schmidlin et al., 2009; Pieper et al., 2013). Many studies have demonstrated that Tripterygium ingredients could inhibit the proliferation and the antibody production of B cells while the molecular mechanisms remained unknown (Tao et al., 1991; Wang and Wu, 1994; Chang et al., 1997; Cascão et al., 2015b). Only one study revealed the molecular mechanisms. Pan etc. found Xinfeng capsule, a proprietary Chinese medicine mainly composed of TwHf, can up-regulate the PTEN level of B cells while down-regulate PDK1 and BAFF/BAFF-R to suppress the activation of PI3K/AKT/mTORC signal pathway and finally inhibit the proliferation and activation of B cells (Pan, 2018). Besides, the levels of related antibodies such as RF, anti-cyclic citrullinated peptide antibody (anti-CCP Ab), IgG, and IgM, were also inhibited. The effects of Tripterygium ingredients on B cells and the molecule mechanisms are summarized in **Table 2**. The molecule mechanisms of B cell are summarized in **Figure 3**.

**Table 2:** The effects of Tripterygium ingredients on B cells.

Subtype	Component		Models	Molecular mechanism	Effects	Animal disease phenotype	Ref.
CD19^+^ B cells	Cel		AIA rats	NA	Decrease the numbers of CD19^+^ cells	Ameliorate	(Cascão et al., 2015a)
CD19^+^CD81^+^ and CD19^+^CD40^+^ B cells		Xinfeng capsule	RA patients	Inhibit PI3K/AKT/mTOR signaling pathway. Up-regulate PTEN; Down-regulate PDK1 and BAFF/BAFF-R	Inhibit B cells proliferation, and activation; Decrease the levels of antibodies, such as RF, anti-CCP Ab, IgG, and IgM	Ameliorate	(Pan, 2018)
B cells		Tripterygium glycosides	B cells	NA	Inhibit the IgG levels secreted by B cell	NA	(Chang et al., 1997)
B cells		Triptolide ethanol extraction	Human peripheral blood	NA	Inhibit B cell proliferation and decrease immunoglobulin levels	Ameliorate	(Tao et al., 1991)
B cells		TwHf	RA patients	NA	Decrease the percentage of B cells	Ameliorate	(Wang and Wu, 1994)

PTEN, Phosphatase and tensin homolog; PDK1, 3-phosphoinositide-dependent protein kinase 1; BAFF, B cell activating factor; BAFF-R, B cell activating factor receptor; PI3K, Phosphatidyl muscle Alcohol-3-kinase; AKT, Serine protein kinase; mTORC, Rapamycin target protein complex; IL, Interleukin; IgG, Immunoglobulin; TwHf, Tripterygium wilfordii Hook f.

## The Role of Tripterygium Ingredients on Macrophage and the Molecule Mechanism

Macrophages are mainly divided into classical activated M1 type and selective activated M2 type. The immuno-inflammatory reaction in RA patients directly affects the polarization of macrophages in peripheral blood, synovium, and synovial fluid, resulting in the continuous increase of M1-type macrophages and disrupting the balance of M1/M2 (Laria et al., 2016). Macrophages also promote RA and participate in the bone destruction of RA through antigen presentation. The degree of synovial macrophage infiltration was positively correlated with the bone destruction and clinical symptoms of RA (Gierut et al., 2010).

**Figure 4:**
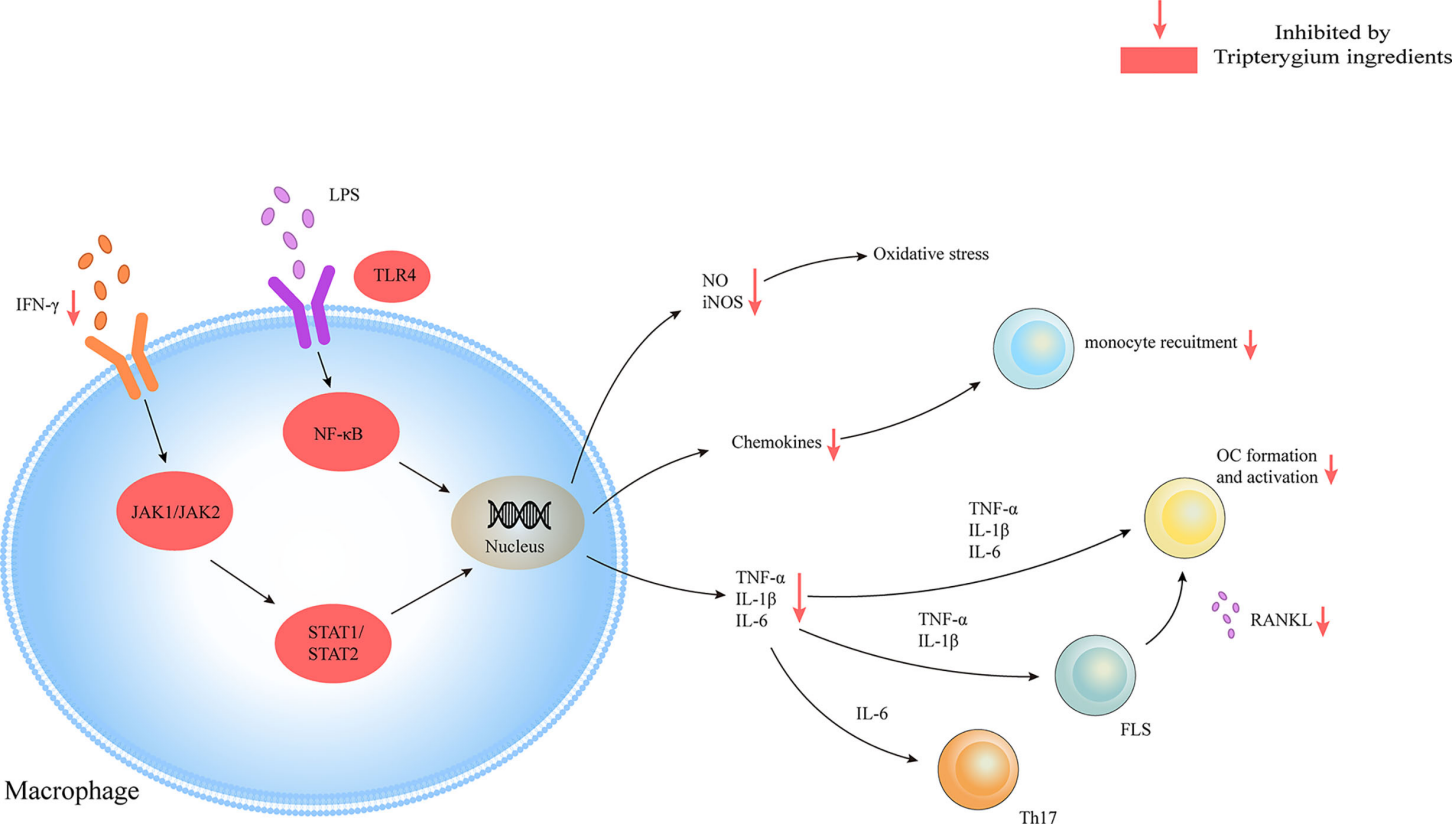
Tripterygium ingredients act on the macrophage. Macrophages play as pro-inflammatory cells in RA with the release of chemokines, cytokines, and oxidative stress molecules through JAK/STAT and TLR/NF-κβ signaling pathways. Tripterygium ingredients block the signaling pathways to lead to the reduction of cytokines, which would inhibit the activation of effector cells. Note: FLS, Fibroblast-like synoviocytes; IFN-γ, Interferon-gamma; IL, Interleukin; JAK, Janus kinase; NF-κβ, Nuclear factor-kappa B; OC, Osteoclast; RANKL, Receptor activator of nuclear factor-κB ligand; STAT, Signal transducer and activator of transcription; TLR, Toll-like receptor; TNF-α, Tumor necrosis factor alpha.

Tripterygium ingredients have been reported to inhibit the M1 polarization and promote the polarization of M2 type macrophages, resulting in the rebalance of the pro-inflammatory and anti-inflammatory cytokines (Feng, 2015; Liu, 2018). Besides, tripterygium ingredients could alleviate synovial macrophage infiltration. However, the molecular mechanism was not explained (Bao et al., 2007; Cascão et al., 2015a; Cascão et al., 2015b; Feng, 2015; Gan et al., 2015; Liu, 2018; Tong et al., 2018; Wang et al., 2018). M1 polarization is mediated by JAK-STAT1 signaling stimulated with IFNγ and characterized by increased iNOS, IL-1β, and TNF-α. Another M1 activated signaling pathway is the TLR4/NF-κB signaling pathway (Lawrence and Natoli, 2011). In the AIA rat model, Cel exerted the anti-inflammatory properties *via* down-regulating the NF-κB and the caspase-1 activation, leading to a decrease of IL-1β and TNF-α secretion in macrophages. Furthermore, the proliferation of Th17 was also inhibited because of lacking cytokines stimulation (Cascão et al., 2012). In addition, research has indicated that Cel blocked the binding of lipopolysaccharides (LPS) to a myeloid differentiation factor2 (MD2) and then inhibited the M1 activation, which was measured by the expression of inflammatory cytokines including TNF-α, IL-6, and IL-1β (Lee et al., 2015). Some researchers (Lin et al., 2001; Ping et al., 2015) also found that Tripterygium ingredients decreased the production of TNF-α, IL-1β, and IL-6 *via* inhibiting the expression of the TLR4, NF-κB, and prostaglandin E2 (PGE2). Besides, NO production and iNOS expression in macrophages were significantly inhibited by Tripterygium ingredients (Wang et al., 2004; Chen et al., 2018). Furthermore, TP inhibited the promoter activity of the iNOS gene and the inducible activity of iNOS transcriptional regulator Oct-1 (Wang et al., 2004). Pyroptosis is a unique and newly discovered mode of programmed cell death, which is triggered by the activation of Caspase-1 (Bergsbaken et al., 2009). It has been found that Cel can inhibit the pyroptosis induced by LPS and ATP *via* inhibiting the enzyme activities of cleaved-Caspase1 and Caspase-1, and finally blocking the secretion of IL-1β in macrophages (Xin et al., 2018). The effect of tripterygium ingredients on macrophages and the molecule mechanisms are summarized in **Table 3**. The figure for the molecule mechanism of macrophages is available in **Figure 4**.

**Table 3:** The role of Tripterygium ingredients on macrophages.

Subtype	Component	Models	Molecular mechanism	Effects	Animal disease phenotype	Ref.
M1, M2 macrophages	Cel	Healthy mice	NA	Inhibit abdominal macrophages to M1 polarization. Promote M2 macrophages polarization	Alleviate	(Liu, 2018)
M1, M2 macrophages	TP	PBMCs, isolated from healthy people, cultured in different pH RPMI-1640	NA	Decrease M1 macrophages level and promote M2 macrophages level	NA	(Feng, 2015)
CD68^+^ CD168^+^ synovial macrophage	Cel	AIA rats	NA	Inhibit the infiltration and proliferation of CD68^+^ CD168^+^ synovial macrophages in the synovial membrane	Alleviate	(Cascão et al., 2015a)
OCP	TP	TNF transgenic mice	NA	Promote the apoptosis of OCP. Inhibit OC proliferation, bone resorption and pro- inflammatory cytokines levels secreted by macrophages	Alleviate	(Wang et al., 2018)
BMDMs	Cel	LPS-induced BMDMs.	Inhibit TLR4 activation *via* prohibiting the binding of LPS to the TLR4/MD2 complex	Inhibit pro-inflammatory cytokine levels and TLR4 activation in macrophages	NA	(Lee et al., 2015)
Macrophage	Cel	E. coli stimulated THP-1 macrophage-like cell line and AIA Wistar rats	Inhibiting activation of NF-κB and caspase-1	Inhibit the release of IL-1β and TNF from macrophages, reducing joint the infiltration and proliferation of TH17 cells	Alleviate	(Cascão et al., 2012)
Macrophage	TP	RAW 264.7 and U937 macrophage-like cell lines	Induce the degradation of Bcl-2 and the activation of caspase-3	Promote macrophages apoptosis	NA	(Bao et al., 2007)
Macrophage	Cel	CIA DBA/1J mice and RANKL induced RAW264.7 cells	Decrease serum TRAP 5b and the expression of osteoclastic genes (Trap, Ctsk, Ctr, MMP-9) and transcriptional factors (c-Fos, c-Jun and NFATc1); Inhibit NF-κB and MAPK	Decrease the infiltration of osteoclast cells in joints. Decrease serum TRAP 5b and the expression of osteoclastic genes and transcriptional factors	Alleviate	(Gan et al., 2015)
Macrophage	Tripterygium glycosides	CFA-induced arthritis rat and LPS-induced RAW264.7	NA	Ameliorate in paw swelling perimeter, arthritics score, and body weight loss. Reduce the levels of inflammatory cytokine (TNF-α, IL-6, and IL-1β) secreted by macrophages	Alleviate	(Tong et al., 2018)
Macrophage	Tripterygium glycosides	LPS-induced RAW264.7	down-regulate the expression of TLR4and NF-κB p65	Decrease the levels of TNF-α and IL-1β secreted by macrophages	NA	(Ping et al., 2015)
Macrophage	TP	LPS induced J774A.1 macrophage and IL-1α induced human synovial fibroblasts	Inhibit COX-2 in macrophages and pro-MMPs 1 and 3 in synovial fibroblasts. Up-regulate TIMPs 1 and 2 levels in synovial fibroblasts	Decrease PGE2 *via* inhibiting COX-2. Inhibit pro-MMPs and Up-regulate TIMPs	NA	(Lin et al., 2001)
Macrophage	Tripterygium wilfordii extraction	LPS-induced RAW264.7	NA	Reduce the production of NO and iNOS mRNA in macrophages	NA	(Chen et al., 2018)
Macrophage	TP and TwHf ethyl acetate extraction	Peritoneal macrophages isolated from AIA C57BL/6J mice	Inhibit NO production and iNOS mRNA expression in macrophages. Inhibit the promoter activity of iNOS gene to regulate its transcript factor (Oct-1) activity	Inhibit the production of NO, iNOS, and the activity of Oct-1	Alleviate	(Wang et al., 2004)
Macrophage	Cel	RAW264.7	Decrease the expression of cleaved-caspase-1 and inhibit caspase-1 enzyme activity	Ameliorate cell pyroptosis	NA	(Xin et al., 2018)

TP, Triptolide; Cel, Celastrol; OC, Osteoclasts; TLR4, Toll-like receptor 4; NF-κB, Nuclear factor activated B cell kappa light chain enhancer; Caspase, Aspartic acid protease containing cysteine; NO, Nitric oxide; OCP, Osteoclast progenitor cells; MD2, Medullary differentiation factor2; PGE2, Prostaglandin E2: Cox, Cyclooxygenase; TIMPs, Metalloproteinases; proMMP, pro-matrix metalloproteinase; TRAP, Tartrate-resistant acid phosphatase; MAPK, Mitogen-activated protein kinases; BMDMs, Bone marrow-derived primary macrophages.

## The Effects of Tripterygium Ingredients on Dendritic Cells (DCS)

TwHf is reported to inhibit DC development and induce DC apoptosis, finally decreasing the DC number, which resulted in the blocking of the naïve T cell activation and ultimately reduced the differentiation of the autoinflammatory T cells (Wang et al., 2001; Chen et al., 2006; Sun, 2017). Also, TP inhibits DC-related chemokines and reduces the sharing of DCs with MHC molecules and co-stimulatory factors of T and B cells, thereby blockade T and B cell activation. Antigenic peptide on MHC molecules, co-stimulatory molecules (CD80, CD86), and IL-12 of DCs promote the differentiation of Th1 cells, which produce IFN-γ and IL-2, required for cell-mediated immunity. Th1 cells directly modulate B cell differentiation into plasma cells. Besides, DCs also mediate the proliferation of these antibody-producing cells by producing BAFF (Khan et al., 2009). Unfortunately, the specific molecule mechanisms were not involved in these studies. Tripterygium ingredients for DCs are summarized in **Table 4**.

**Table 4:** The role of Tripterygium ingredients for DCs.

Component	Models	Molecular mechanism	Effects	Animal disease phenotype	Ref.
Duantengyimu decoction	GM-CSF and IL-4 stimulate PBMCs isolated from patients with RA	NA	Decrease the expression of CCR5 and CCR7, decrease the secretion of CXCL9 and CXCL10, and inhibit the migration of DC	NA	(Sun, 2017)
Tripterygium Wilfordii Saponins	GM-CSF, TNF-α, and IL-4 stimulate PBMCs	NA	Inhibit the expressions HLA-DR and CD80 on the membrane. Decrease the synthesis of IL-12 p40 subunit	NA	(Wang et al., 2001)
Tripterygium glycosides	DCs isolated from rats	NA	Reduce the expression of MHC-II, CD80, CD86, and CD40 on the membrane of DC	NA	(Chen et al., 2006)

TP, Triptolide; Caspase, Aspartic acid protease containing cysteine; CCR, Chemokine receptor; CXCL, C-X-C motif chemokine ligand; MAP, Mitogen activated protein; MHC, Major histocompatibility complex.

## The Functions of Tripterygium Ingredients on Osteoclasts (OCS) and the Molecule Mechanism

**Figure 5:**
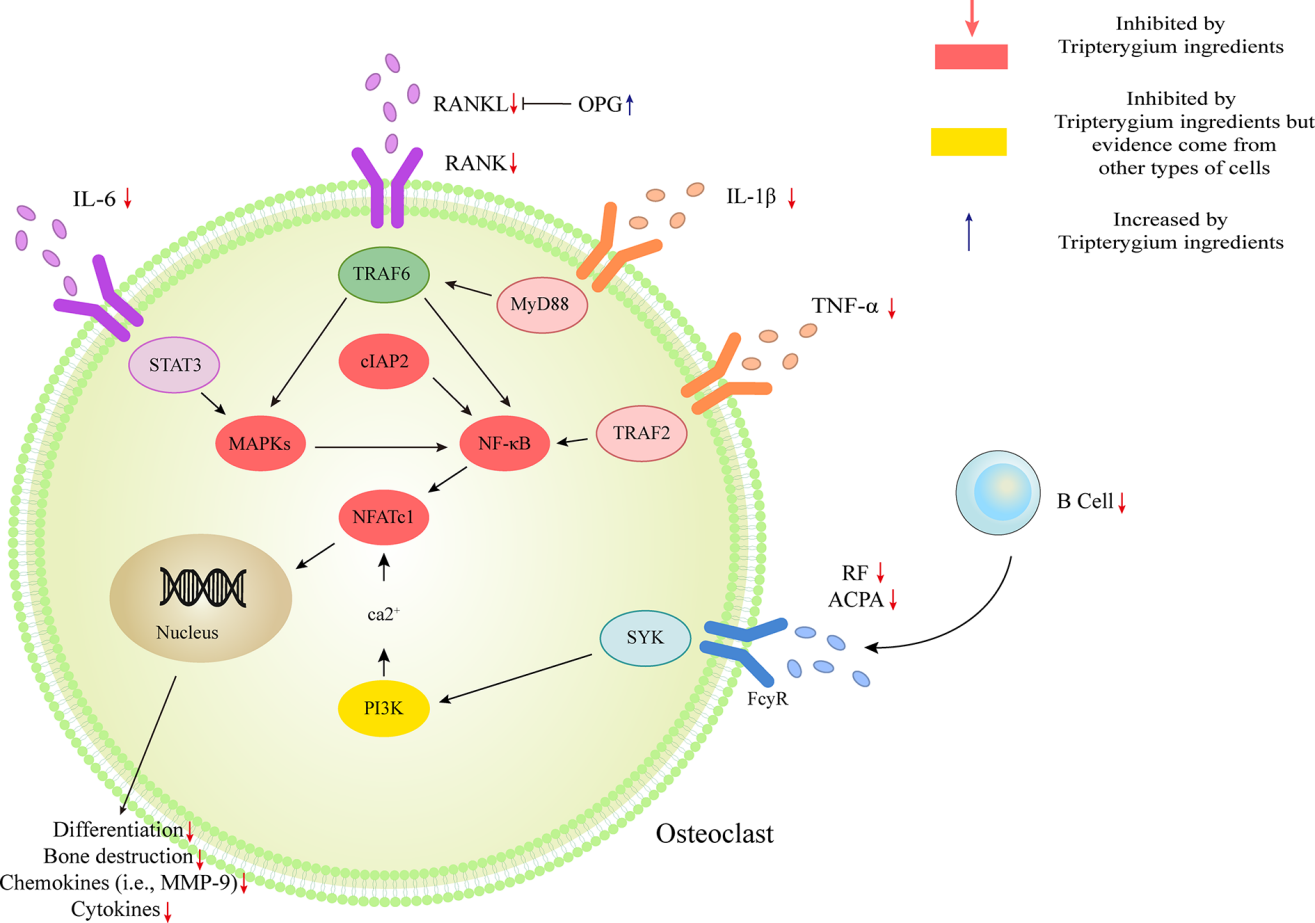
Tripterygium ingredients act on osteoclast. The activation of osteoclast mostly involved IL-1β/Myd88/TRAF6/NF-κB, TNF-α/TRADD/TRAF2/NF-κB, IL-6/STAT3/MAPK, and RANKL/RANK signal transduction. Besides, the anti-bodies released by B cells would promote the activation of osteoclast *via* PI3K signaling. OPG takes part as a decoy receptor for RANKL and inhibiting RANKL-RANK binding. By rebalancing the RANKL and OPG levels, and inhibiting the pathways mentioned above, tripterygium ingredients negatively regulate the differentiation, bone destruction, cytokines and chemokines expression of osteoclast. Note: ACPA, Anti-citrullinated protein antibodies; cIAP2, Cellular inhibitor of apoptosis 2; IL, Interleukin; MAPK, Mitogen-activated protein kinase; MYD88, Myeloid differentiation primary response 88; NFATc1, Nuclear factor of activated T cells 1; RANK, Receptor activator of nuclear factor-κB; RANKL, Receptor activator of nuclear factor-κB ligand; RF, Rheumatoid factor; STAT, Signal transducer and activator of transcription; SYK, Spleen tyrosine kinase; TNF-α, Tumor necrosis factor-alpha; TRAF, Tumor necrosis factor receptor-associated factor.

OC-mediated bone resorption is one of the typical manifestations of RA. OCs, giant multinucleated cells derived from the monocyte lineage, are the only cells capable of resorbing bone (Teitelbaum and Ross, 2003). Receptor activator of nuclear factor kappa-B (RANK)/Receptor activator of nuclear factor kappa-B ligand (RANKL)/Osteoprotegerin (OPG) is the most crucial pathway of OC differentiation. Leibbrandt A etc. has demonstrated that RANKL is the critical mediator of OC activation and joint destruction; In a rat model of arthritis, osteoblasts and bone marrow stromal cells produce RANKL, which then triggers local development and activation of OCs. This finding has now become the basis for osteoimmunology (Leibbrandt and Penninger, 2009). Multiple studies have confirmed that Tripterygium ingredients can inhibit the expression of RANK and RANKL, thereby increasing the proportion of OPG, which can antagonize the function of RANK and finally inhibit the differentiation of OCs and reduce bone destruction (Nanjundaiah et al., 2012; Feng et al., 2013; Liu et al., 2013; Wang, 2015). Youn-Kwan Jung et al. (Jung et al., 2019) reviewed the roles of inflammatory signal pathways, including IL-1β/Myd88/TRAF6/NF-κB, TNF-α/TRADD/TRAF2/NF-κB, IL-6/STAT3/MAPK, and RANKL/RANK signal transduction. In these inflammatory pathways, TwHf or its active components impaired the release of cytokines/chemokines, reduced osteoclast differentiation, and activation, then finally blocked bone erosion in mice with collagen-induced arthritis through inhibiting the phosphorylation of NF- κB p65, MAPK (ERK, JNK, and p38) and reducing the expression of transcription factors c-Fos, c-Jun, and NFATcl (Gan, 2013; Qian et al., 2015). TwHf or its active components can also promote the apoptosis of OCs and osteoclast precursor (OCP) (Wang et al., 2018; Wang S. et al., 2019). The mechanism may be due to the inhibition of cIAP2 (the positive regulatory protein of TNF and NF-κB signaling pathway). Furthermore, TP has been reported to block OC differentiation by down-regulating the receptor for advanced glycation end-products (RAGE) and the high-mobility group box chromosomal protein 1 (HMGB1) (Wang et al., 2017). RAGE and its ligands (i.e., HMGB1) are necessary for the skeletal homeostasis and related-disease onset/progression (Plotkin et al., 2019). The elevated levels of RAGE and HMGB1 induce osteoblast apoptosis and OC differentiation/activity. Tripterygium ingredients on OC and the molecule mechanisms are summarized in **Table 5**. The figures for the molecule mechanism of OCs are available in **Figure 5**.

**Table 5:** The function of Tripterygium ingredients on OC.

Component	Models	Molecular mechanism	Effects	Animal disease phenotype	Ref.
Cel	CIA mice	Reduce RANKL levels	Inhibiting OC differentiation	Alleviate	(Wang, 2015)
TP	CIA mice	Regulating the RANKL/RANK/OPG signaling pathway	Inhibiting OC differentiation	Alleviate	(Liu et al., 2013)
Cel	AIA Lewis rats and RANKL induced RAW264.7	Decrease RANKL levels and regulate RANKL/OPG ratio	Reduce OC proliferation. Ameliorate bone destruction. Decrease levels of upstream pro-inflammatory cytokine (i.e., IL-6) and downstream effectors (i.e., MMP-9)	Alleviate	(Nanjundaiah et al., 2012)
Cel	IL-1β stimulated MH7A	Decrease RANKL levels and increase OPG levels	Inhibit OC differentiation and activation	NA	(Feng et al., 2013)
Cel	RANKL induced RAW264.7 and CIA mice	Inhibit the protein phosphorylation of RANK downstream signalings, such as NF-κB p65, MAPK (ERK, JNK, p38) and the expression of the relevant transcription factors (i.e., c-Fos, c-Jun, and NFATcl)	Inhibit OC differentiation and bone resorption	Alleviate	(Gan, 2013)
Cel	RANKL induced RAW264.7	NA	Inhibit OC differentiation and chemokine CCl4	NA	(Qian et al., 2015)
TP	C57BL/6 mice bone marrow mesenchymal stem cells induced by RANKL, M-CSF, and HMGB1	Reduce the expression of RAGE mRNA to inhibit HMGB1	Inhibit OC differentiation	NA	(Wang et al., 2017)
TP	Co-cultures system of Tregs and BMMs	NA	Increase the levels of IL-10 and TGF-β1 secreted by Treg to inhibit OC differentiation and bone resorption	NA	(Xu, 2016; Xu et al., 2016)
TP	TNF-Tg mice and spleen cells isolated and induced to differentiate into OCs by M-CSF	Down-regulate the cIAP2	Promote OCP apoptosis and OC reduction	NA	(Wang S. et al., 2019)
TP	TNF-Tg mice	NA	Promote apoptosis rates of OCP and OC. Prohibit the bone erosion	Alleviate	(Wang et al., 2018)

TP, Triptolide; Cel, Celastrol; RANKL, Nuclear factor kappa B ligand receptor activator; RANK, Nuclear factor receptor activator; OPG, Osteoprotegerin; NF-kappa B, Nuclear factor activated B cell κ light chain enhancer; MAPK, Mitogen activated protein kinase; ERK, Extracellular regulatory protein kinase; JNK, Jun N-terminal kinase; NFATcl, Osteoclast activated T nuclear factor 1; CCl4, C-C motif chemokine ligand 4; HMGB1, High mobility group protein B1; RAGE, Receptor for advanced glycation end products; OC, Osteoclasts.

## The Role of Tripterygium Ingredients on FLS and the Molecule Mechanism

Synovial inflammation and synovial cell hyperplasia is a distinctive feature of RA. Synovial cells are composed of two types of cells, including type A and type B. Type A cells have a phagocytic function and are macrophage-like cells; type B cells are fibroblast-like, called FLS (Junqueira and Mescher, 2013). FLS is abundant in the endoplasmic reticulum and can secrete protein complexes (mucin) and hyaluronic acid in synovial fluid. FLS contributes mainly to the exacerbation of RA by attaching to, followed by invading into, and finally degrading cartilage and bone (Lefèvre et al., 2009). FLS are the primary cells leading to joint destruction in RA (Bartok and Firestein, 2010).

The molecular pathologic basis RA-FLS includes the MAPK and NF-κB pathways. These pathways are the most widely studied to mediate the aggressiveness of FLS in RA (Bottini and Firestein, 2013; Ganesan and Rasool, 2017). NF-κB pathway, a significant regulator of pro-inflammatory cytokine production, activates NF-κB kinase (IKK) subunit β (IKKβ) in the cytosol through IL-1β, TNF-α, and TLR signaling. The activation of IKKβ results in the NF-κB family inhibitor proteins (IκB) degradation, promoting NF-κB to migrate freely into the nucleus and initiate gene transcription (Bottini and Firestein, 2013). Cel inhibited the translocation of NF-κβ p65 and reduced the phosphorylation of IKBα and IKK in FLSs from patients with RA, resulting in the decreased expression of several chemokines (i.e., CCR2, CXCR4, CCL2, CXCL10, and CXCL12), cytokines (i.e., IL-6, IL-8, and MCP-1), and matrix metalloproteinase-9 (MMP-9) (Fang et al., 2017). Besides, HIF-1α binding to the CXCR4 promoter would increase the transcriptional activity of CXCR4, consequently leading to FLS migration and invasion. However, it could be reversed by Cel treatment (Li et al., 2013b). Guo et al. (Li et al., 2012) found that Cel inhibited IκBα phosphorylation and nuclear translocation of NF-κB. Cel also has been found to inhibit the expression of MMP-9 by suppressing the binding activity of NF-κB to the MMP-9 promoter (Li et al., 2012; Li et al., 2013a). Furthermore, MMP-9 suppression was also related to the inhibition of the TLR4/MyD88/NF-κB pathway (Li et al., 2013a). As a result, Cel changes the phenotype of FLS migration and invasion *via* the molecule mechanism mentioned above. RA-FLS releases important inflammatory cytokines (TNF-α, IL-1β, IL-6, IL-21, IL-22, and IL-32), chemokines (CXCL1, CXCL5, MCP-1, G-CSF, and IL-8) and Inflammatory mediators (TLR-2, TLR-3, TLR-4, iNOS, and COX-2), which promotes the infiltration of monocytes, macrophages, neutrophils, DCs, T cells, and B cells into joints and results in chronic inflammation and joint destruction (Bottini and Firestein, 2013; Ganesan and Rasool, 2017). Additionally, TP also was found to reduce the FLS migration and invasion by targeting JNK/MAPK signaling pathway (Yang et al., 2016). Moreover, LLDT-8, a Tripterygium derivative, decreased the secretion of chemokines in FLS (Ping et al., 2016; Jia et al., 2017).

**Figure 6:**
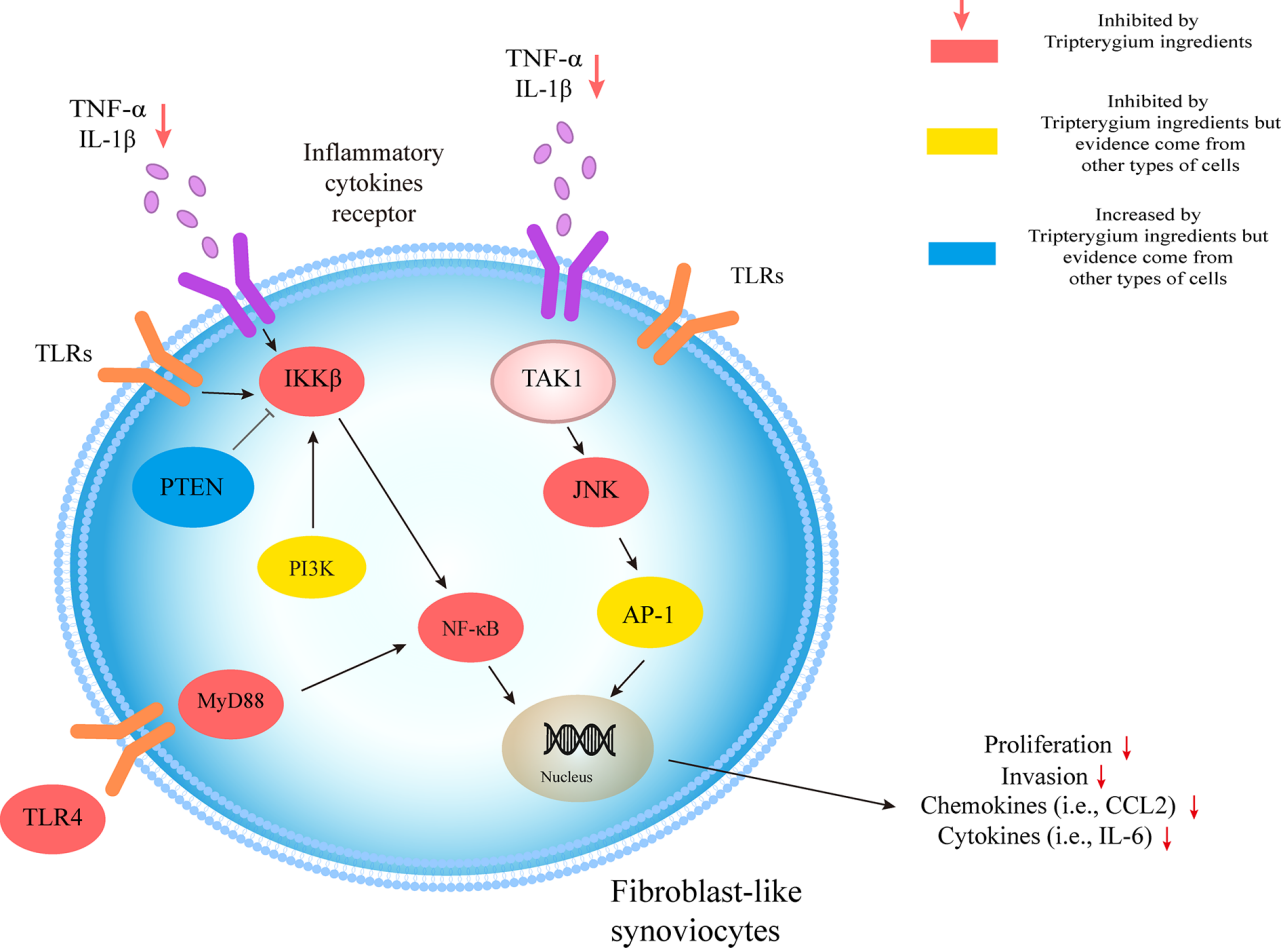
Tripterygium ingredients act on FLS. The molecular pathologic basis FLS includes the MAPK and NF-κB pathways. By prohibiting the signal transduction in the pathways, tripterygium ingredients alleviate the proliferation, invasion of FLS, which would promote bone erosion in RA. PTEN and PI3K, which inhibit and promote the activity of IKKβ, respectively, also are related in the networks of FLS. We suppose tripterygium ingredients could increase PTEN and decrease PI3K to alleviate the pathogenicity of FLS. Note: AP-1, Activator protein 1; FLS, Fibroblast-like synoviocytes; JNK, c-Jun N-terminal kinase; IL, Interleukin; IKKβ, IκB Kinase β; MYD88, Myeloid differentiation primary response 88; NF-κβ, Nuclear factor-kappa B; PI3K, Phosphoinositide 3-kinase; PTEN, Phosphatase and tensin homolog; TNF-α, Tumor necrosis factor-alpha; TLR, Toll-like receptor; TAK1, Transforming growth factor beta-activated kinase 1.

Many studies showed the Tripterygium ingredients have the properties to promote FLS apoptosis and cell cycle arrest and inhibit FLS autophagy (Xu, 2013; Xu et al., 2013; Lei et al., 2015; Su et al., 2017; Wong et al., 2019). It may be relevant to the increased expression of Bax/Bcl-2, Caspase-3, Caspase-9, and regulating by Ca2+/calmodulin-dependent protein kinases beta (CaMKK)-AMPK-mTOR signaling pathway. Tripterygium ingredients for FLS and the molecule mechanism are summarized in **Table 6**. The figures for the molecule mechanism of FLS are available in **Figure 6**.

**Table 6:** The function of Tripterygium ingredients on FLS.

Component	Models	Molecular mechanism	Effects	Animal disease phenotype	Ref.
Cel	FLSs from patients with RA	Reduce the phosphorylation of IKK and IKBα, and inhibit the translocation of NF-κβ p65 from the cytoplasm to the nucleus	Inhibit FLS proliferation and invasion. Reduce the levels of FLS pro-inflammatory cytokines (i.e., IL-6, IL-8, MCP, and MMP9) and chemokines (i.e., CCL2, CXCL10, CXCL12, CCR2, and CXCR4)	NA	(Fang et al., 2017)
Cel	AIA model	Inhibited the transcriptional activity of MMP-9 by suppression of the binding activity of NF-κB in the MMP-9 promoter, and inhibited IκBα phosphorylation and nuclear translocation of NF-κB	Suppressed the IL-17A-induced migration and invasion abilities of FLS	Alleviate	(Li et al., 2012)
Cel	FLSs isolated from the synovium of active RA patients	Inhibit the transcriptional activity of MMP-9 and TLR4/MyD88/NF-κB signaling pathway	Inhibit FLS invasion and migration	NA	(Li et al., 2013a)
TP	FLSs isolated from active RA patients and CIA DBA/1 mice	Inhibit JNK/MAPK signaling pathway	Inhibit FLS invasion and migration	Alleviate	(Yang et al., 2016)
Cel	FLSs isolated from active RA patients	Inhibit the binding activity of HIF-1α in the CXCR4 promoter to inhibit the transcription activity of CXCR4	Inhibit FLS invasion and migration	NA	(Li et al., 2013b)
TP	MH7A cell line	NA	Promote MH7A cell apoptosis; Decrease the levels of IL-1β, IL-6, and IL-8; Induce membrane ultrastructural changes	NA	(Su et al., 2017)
Cel	Human fibroblast-like synoviocytes-rheumatoid arthritis cells	Increase the expression of Bax/Bcl-2 and promote proteolytic cleavage of Caspase-3, Caspase-9, and PARP	Lead to FLS DNA damage and cycle arrest; Promote FLS apoptosis	NA	(Xu, 2013; Xu et al., 2013)
Cel	Immortalized wild-type and Bax-Bak double-knockout mouse embryonic fibroblasts; RASFs isolated from RA patients; AIA rats	Inhibit SERCA to induce autophagy-dependent cytotoxicity in RASFs/RAFLS *via* Ca2^+^/calmodulin-dependent kinase kinase-β-AMP-activated protein kinase-mTOR pathway	Induce autophagic FLS death in RASFs/RAFLS	Alleviate	(Wong et al., 2019)
TP	MH7A cell line	NA	Inhibit angiogenesis	NA	(Zhang et al., 2008; Mao et al., 2009)
TP	FLSs isolated from RA patients	NA	Lead to FLS cycle arrest and promote FLS apoptosis	NA	(Lei et al., 2015)
LLDT-8	FLSs isolated from RA patients	NA	Inhibit FLS cytokines and chemokines (i.e., IL-6, CCL3, and CCL5)	NA	(Ping et al., 2016; Jia et al., 2017)

TP, Triptolide; Cel, Celastrol; NF-κB, Nuclear factor activated B cell kappa light chain enhancer; Caspase, Aspartic acid protease containing cysteine; TLR4, Toll-like receptor 4; JNK, Jun N-terminal kinase; MAPK, Mitogen activated protein kinase; mTOR, Rapamycin target protein; MMP-9, Matrix metalloproteinase-9; IL, Interleukin; AMPK, AMP-dependent protein kinase.

## Discussion

**Figure 7:**
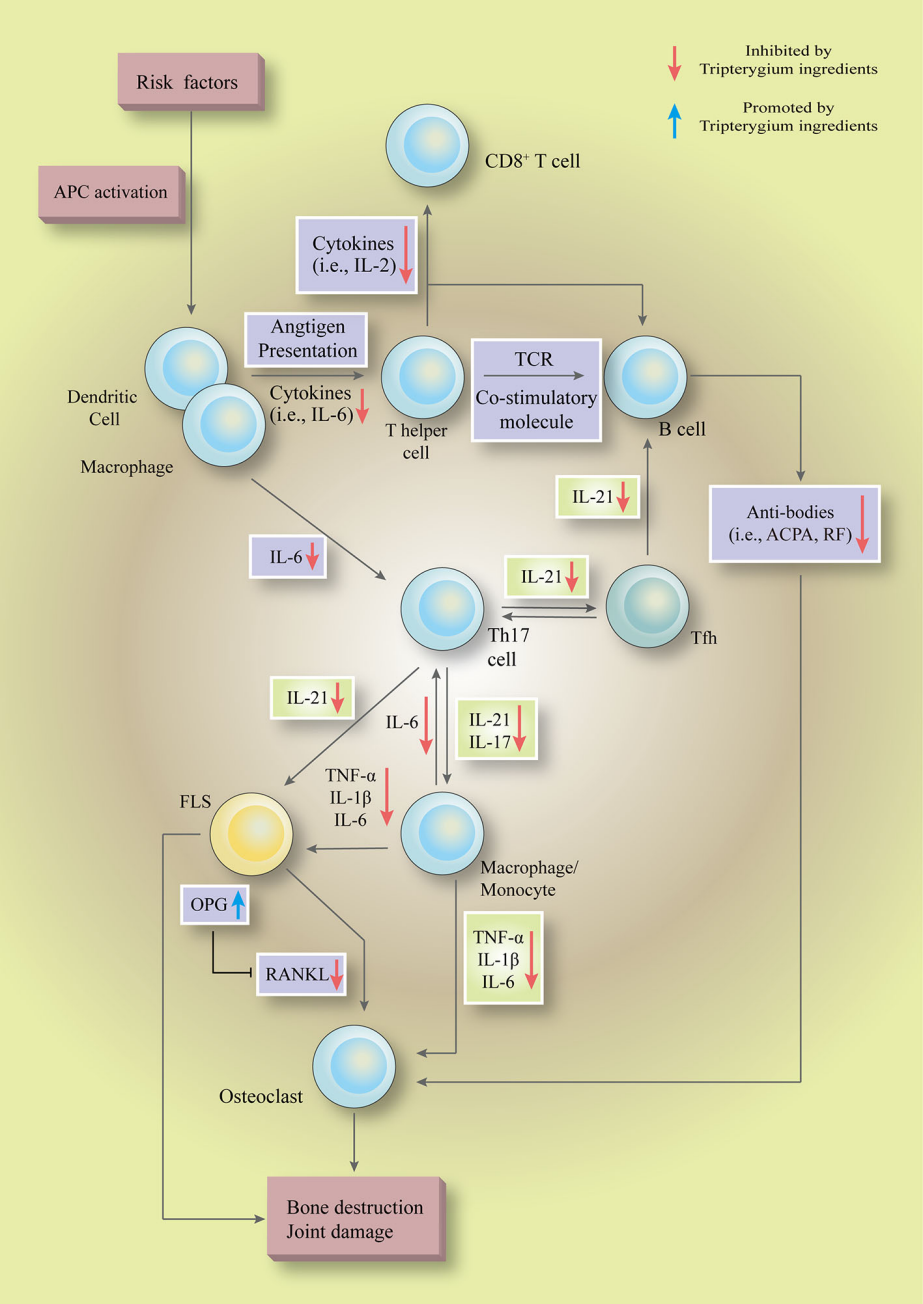
Tripterygium ingredients could inhibit multiple pathways, such as the NF-κB pathway, JAK-STAT pathway, and PI3K-mTOR pathway, to regulate the hyperactive as well as pathogenicity biological functions in a various type of cells ( to ). In the initial phase, tripterygium ingredients inhibit the immunological recognition functions of APCs to block the pathogenicity signals which are responsible for activating the lymphocytes. In the secondary stage, tripterygium ingredients could inhibit the humoral immunity and cellular immunity of lymphocytes. Meanwhile, the pro-inflammatory signals are amplified by pro-inflammatory cells, such as macrophages. The pro-inflammatory cells release inflammatory cytokines and chemokines to recruit and activate immune cells (i.e., APCs and T cells), connective tissue cells (i.e., macrophages and FLS) and OCs to infiltrate into the joints. Besides, the pro-inflammatory cells lead to a systemic inflammatory response, further promoting the pathogenicity signals of lymphocytes in central and peripheral immune organs. Tripterygium ingredients prohibit the pro-inflammatory signals, alleviate the infiltration and activation of pathogenicity cells in the joint, and finally interrupt the vicious circle which formed by pro-inflammatory cells and immune cells. In the final stage, tripterygium ingredients relieve the joint damage and bone destruction by mediating the expression of OPG and RANKL. Note: APC, Antigen-presenting cell; ACPA, Anti-citrullinated protein antibodies; FLS, Fibroblast-like synoviocytes; IL, Interleukin; OPG, Osteoprotegerin; RANKL, Receptor activator of nuclear factor-κB ligand; RF, Rheumatoid factor; TCR, T-cell receptor; TNF-α, Tumor necrosis factor-alpha.

We systematically summarized the role of tripterygium ingredients in the RA treatment as well as explained the therapeutic mechanism (**Figure 7**). The NF-κB pathway is a common pathway involved in TwHf-treated RA. It has been involved in mediating multiple genes, such as the genes of cytokines (i.e., IL-6, IL-17, and TNF-α), chemokines (i.e., CCL2 and CXCL5), growth factors (i.e., GM-CSF and M-CSF), regulators of apoptosis (i.e., Bcl-2) and transcription factors (i.e., HIF-1α), to regulate cell function, cell death and survival, and proliferation (Mitchell and Carmody, 2018). Experimental inhibitors targeted the IKK kinases to inhibit the activation of NF-κB, but they failed due to toxicity in genetic models (Mitchell and Carmody, 2018). The failure indicated that a broad blockade of NF-κB activation maybe an impracticable approach. Thus, some drugs focus on the non-canonical NF-κB pathway in RA, such as BAFF/NF-κB, RANK/NF-κB signaling (Noort et al., 2015). A phase II trial showed that belimumab [a biologics target B lymphocyte stimulator (BLyS)] was efficacy and well-tolerated in patients with RA (Stohl et al., 2013). Denosumab is a monoclonal antibody neutralizing RANKL. Up to date, many clinical studies have demonstrated that denosumab could inhibit the progression of joint destruction and increase bone mineral density, including a double-blind, placebo-controlled phase 3 trial (Deodhar et al., 2010; Dore et al., 2010; Sharp et al., 2010; Takeuchi et al., 2016; Yue et al., 2017; Takeuchi et al., 2019). However, none of the studies reported there is any benefit in improving disease activity. It is also regrettable that none of the studies test the expression of cytokines, chemokines, and RA-related pathogenicity cells. NF-κB pathways, including canonical and non-canonical pathways, are critical targets of tripterygium ingredients. In the experimental and clinical dimensions, these could explain why tripterygium ingredients could reduce the levels of many chemokines, cytokines, and growth factors in different cells to improve disease activity as well as inhibit the progression of joint destruction. TwHf and its ingredients could be regarded as one of DMARDs. Thus, they are widely used in treating RA in China. The last 24 weeks, open-label, multicentre, randomized controlled trial demonstrated MTX+TwHF was better than MTX monotherapy (Lv et al., 2015). Furthermore, three meta-analyses (the trial mentioned above included) showed that MTX+TwHF had advantages in improving the laboratory index (CRP, RF, ESR), clinical symptoms, and clinical efficacy, compared with MTX alone (assessed in ACR20, ACR50, and ACR70) (Li et al., 2019; Wang X. et al., 2019; Chen et al., 2020). Another small sample meta-analyses showed that TwHF could decrease bone destruction scores (Zhu et al., 2019).

Our previous research using a bioinformatics approach demonstrated that Kunxian Capsule (a Traditional Chinese Medicine (TCM) patent prescription mainly comprises Levl. Hutch) could target at PI3K/AKT/mTOR signaling pathway (Tang et al., 2020). Therefore, we speculate that some proteins in the PI3K-AKT-mTOR signal pathway are the most likely direct targets of tripterygium ingredients. The signaling pathway is an intracellular signaling pathway and performs multiple physiological functions, such as regulating the cell cycle, survival, and growth (Yap et al., 2008; Ersahin et al., 2015). By far, no clinical study has reported PI3K inhibitors approved by the FDA (idelalisib, copanlisib, duvelisib, and alpelisib) in treating RA. Some drugs were demonstrated they were effective in treating RA models *via* PI3K signaling pathway *in vivo* and *in vitro*, including one PI3K inhibitor (Boyle et al., 2014; Feng and Qiu, 2018; Qi et al., 2019).

The two pathways mentioned above have a variety of biological functions. Both of them control cell death, survival, and proliferation. We found that the therapeutic effects of tripterygium ingredients are mostly related to the reduction of the absolute number of cells. At the same time, there are some differences in stimulating signals, signal receptors, and transcription factors required for cascade reactions in different cells, which is related to the multi-target of TwHf. Considering the adverse effects (AEs) of these drugs, a variety of healthy cells in multiple systems (i.e., liver cells) are also be affected. Therefore, the AEs of tripterygium ingredients could be due to the inhibition of NF-κB and PI3K pathways. Ameliorating AEs through drug matching may be a feasible strategy (Tang et al., 2020). For example, some Chinese researchers matched the TwHf with *Cistanche deserticola Ma* or *Cuscuta chinensis Lam*, to reduce the reproductive toxicity of TwHf (Dong et al., 2009; Jing and He, 2013). Nevertheless, whether drug matching would impair the curative effect, still needed to be discovered.

There are many deficiencies in this review. The studies on the drugs/ingredients are all indirect mechanism studies, even with only cell phenotypes but no specific molecular mechanism. Besides, most of them are normal phenotypes in this field, such as inhibition of the apoptosis and differentiation of T cells or impaired proliferation, migration, and invasion of FLS. Moreover, none of the studies analyzed the direct interaction between the drugs and proteins *via* bioinformatics and mass spectrometry approaches. For example, computer simulation and electrospray mass spectrometry (ESI-MS) were used to explore the inhibitory effect of paclitaxel and aryl ether ketone on farpentine diphosphate synthetase by binding to isoprene diphosphate site (Liu et al., 2014), and explain the anticancer and anti-infective drug mechanism of paclitaxel and aryl et her ketone in the direct interaction mechanism. Therefore, we could follow the methods which were used in the study, as mentioned above. For example, we could use bioinformatics to identify whether TP and Cel could bind to some sites of PI3K, ATK, and NF-κB. Subsequently, we could use ESI-MS to validate it.

## Author Contributions

YT: evidence collection, manuscript preparation and write the central part of the manuscript. QL: evidence collection and manuscript editing. FY: evidence collection and write the minor part of the manuscript. YiZ: evidence collection, figures preparation. XZ: gave many professional suggestions and project funding. CW: ideas, reviewed the manuscript, and project funding. YuZ: critically reviewed the manuscript, study initiation, and project funding. All authors contributed to the article and approved the submitted version.

## Funding

Research was funded by National Key R&D Program of China (2018YFC1705500) to CW, National Natural Science Foundation of Zhejiang Province (No.LY20H270007), TCM Science and Technology Plan of Zhejiang Province (No.2020ZQ012) to YZ, and National Natural Science Foundation of China (81673623) to ZX.

## Conflict of Interest

The authors declare that the research was conducted in the absence of any commercial or financial relationships that could be construed as a potential conflict of interest.
