# Supplementary material for: Tripterygium Ingredients for Pathogenicity Cells in Rheumatoid Arthritis
Source: Front Pharmacol. 2020 Oct 2;11:583171. doi: 10.3389/fphar.2020.583171 (PMC7567162; doi:10.3389/fphar.2020.583171)
Supplement: Supplementary file 1 [file Table_1.docx]

# To locate disease (via MEDLINE)

#1. Rheumatoid Arthritis[Title/Abstract]

#2. "Arthritis, Rheumatoid"[Mesh]

#3. #1 or #2

# To locate cell (via MEDLINE)

#4. "T-Lymphocytes"[Mesh]

#5. "T-Lymphocytes, Helper-Inducer"[Mesh]

#6. "Th17 Cells"[Mesh]

#7. Th1[Title/Abstract]

#8. Th2[Title/Abstract]

#9. Th17[Title/Abstract]

#10. T cell[Title/Abstract]

#11. T helper cell[Title/Abstract]

#12. T follicular helper cells[Title/Abstract]

#13. Tfh[Title/Abstract]

#14. #4 or #5 or #6 or #7 or #8 or #9 or #10 or #11 or #12 or #13

#15. "B-Lymphocytes"[Mesh]

#16. B cell[Title/Abstract]

#17. Plasma cell[Title/Abstract]

#18. #15 or #16 or #17

#19. "Macrophages"[Mesh]

#20. Macrophage[Title/Abstract]

#21. RAW264.7[Title/Abstract]

#22. THP-1[Title/Abstract]

#23. U937[Title/Abstract]

#24. #20 or #21 or #22 or #23

#25. "Dendritic Cells"[Mesh]

#26. "Antigen-Presenting Cells"[Mesh]

#27. Dendritic Cell[Title/Abstract]

#28. MUTZ-3[Title/Abstract]

#29. HL-60[Title/Abstract]

#30. #25 or #26 or #27 or #28 or #29

#31. "Osteoclasts"[Mesh]

#32. Osteoclast[Title/Abstract]

#33. 4B12[Title/Abstract]

#34. #31 or #32 or #33

#35. "Connective Tissue Cells"[Mesh]

#36. "Fibroblasts"[Mesh]

#37. fibroblast[Title/Abstract]

#38. MH7A[Title/Abstract]

#39. #35 or #36 or #37 or #38

#40. #14 or #18 or #24 or #30 or #34 or #39

# To locate intervention (via MEDLINE)

#41. "Tripterygium"[Mesh]

#42. Tripterygium[Title/Abstract]

#43. Triptolide[Title/Abstract]

#44. Celastrol[Title/Abstract]

#45. Wilforgine[Title/Abstract]

#46. Wilforine[Title/Abstract]

#47. Demethylzeylasteral[Title/Abstract]

#48. Wilfortrine[Title/Abstract]

#49. LLDT-8[Title/Abstract]

#50 #41 or #42 or #43 or #44 or #45 or #46 or #47 or #48 or #49

# To locate Tripterygium Ingredients for Pathogenicity Cells in Rheumatoid arthritis (via MEDLINE)

#3 AND #40 AND #50
